# Metabolic Syndrome Is Associated With Altered mRNA and miRNA Content in Human Circulating Extracellular Vesicles

**DOI:** 10.3389/fendo.2021.687586

**Published:** 2021-08-12

**Authors:** Yongxin Li, Yu Meng, Xiangyang Zhu, Andre Van Wijnen, Alfonso Eirin, Lilach O. Lerman

**Affiliations:** ^1^Department of Vascular Surgery, The Affiliated Hospital of Qingdao University, Qingdao, China; ^2^Division of Nephrology and Hypertension, Mayo Clinic, Rochester, MN, United States; ^3^Central Laboratory, The Fifth Affiliated Hospital of Jinan University, Heyuan, China; ^4^Department of Nephrology, The First Affiliated Hospital of Jinan University, Guangzhou, China; ^5^Departments of Orthopedic Surgery, Biochemistry and Molecular Biology, Mayo Clinic, Rochester, MN, United States

**Keywords:** metabolic syndrome, RNA sequencing, circulation, extracellular vesicles, extracellular vehicles

## Abstract

As mediators of intercellular communication, circulating extracellular vehicles (EVs) can modulate tissue and cellular pathways by altering transcription profiles in recipient cells, and their content may reflect the status of their parent cells. However, whether their cargo is altered in the metabolic syndrome (Mets) remains unclear. We hypothesized that MetS altered mRNAs and miRNAs packed within circulating-EVs. EVs were collected from plasma of patients with MetS or age-matched Lean controls (n=4 each). RNA sequencing was performed to identify dysregulated mRNAs and miRNAs, and analyze genes targeted by miRNAs, top pathways, and diseases associated with MetS-EVs. MetS patients showed elevated body weight, blood pressure, glucose, insulin, and liver injury markers levels. 1,446 mRNAs were downregulated and 32 upregulated in MetS- compared to Lean-EVs, whereas 40 miRNAs were selectively enriched and 10 downregulated in MetS-EVs. MetS upregulated in EVs genes involved in apoptosis, mitochondrial regulation, transport, and lipoproteins, but downregulated vessel and heart development, protein complex biogenesis, and angiogenesis. MetS also upregulated miRNAs targeting genes implicated in cellular processes, including oxidation–reduction, and downregulated miRNAs capable of modulating catalytic activity, as well as heart, blood vessel, and skeletal development, transcriptional regulation, apoptosis, and cell cycle. Our study, thus, indicates that human subjects with MetS show modified cargo of circulating EVs, which in turn may modulate several critical cellular functions and fate. These EVs may reflect the anomalous status of their parent cells, and potentially serve as important regulators, biomarkers, and targets in the progression and treatment of MetS.

## Background

The metabolic syndrome (MetS) is a collective cluster of disease risk factors, including dyslipidemia, obesity, inflammation, insulin resistance, and hypertension, affecting numerous people worldwide (1). The presence of MetS significantly increases disease risk for type 2 diabetes, atherosclerosis, cardiovascular, and chronic kidney disease, and ultimately leads to organ injury and dysfunction (2). The pathogenesis of MetS involves activation of a plethora of signaling pathways and potential damage to cell types, tissues, and target organs (3).

Extracellular vesicles (EVs), membrane-bound nanoparticles, can be released from almost every cell type and play an essential role in a wide range of physiological and pathological process *in vivo* (4). Besides apoptotic bodies, the two main types of EVs are exosomes and microvesicles, which are the most studied EVs populations. Known as carriers for circulating miRNAs, plasma EVs also contain other nucleotides, lipids, and proteins (5). EVs function as mediators of intracellular communication by shuttling bioactive cargo between the parent cells in which they originate and neighboring or distant recipient cells (6). By altering transcription profiles in recipient cells, EVs can modulate tissue metabolism and cellular pathways (7). Growing evidence indicates that EVs are prevalent in biofluids, including serum/plasma, saliva, bronchoalveolar lavage fluid, and urine, and that their composition changes in disease states (8), positioning EVs as novel circulating biomarkers (9) that reflect the status of their parental cells.

Our group recently demonstrated that EVs released by pig adipose tissue-derived mesenchymal stem cells (MSCs) are selectively packed with micro-RNAs (miRNAs), mRNAs, and proteins, which possess the capacity to modify selective pathways in recipient cells (10, 11). miRNAs are small non-coding RNAs that regulate gene expression post-transcriptionally and may play a crucial role in the pathophysiology of MetS (12), suggesting them as a new class of endocrine factors (13). Importantly, recent studies have revealed that miRNAs are detectable in the peripheral circulation packed in different types of EVs (14).

Furthermore, we have also shown that MetS can modify the cargo and size of porcine adipose tissue-derived MSC, including mRNAs, miRNAs, and proteins, which might in turn control some important cellular functions (15, 16). In addition, the transcriptome and proteome of particularly genes in MSCs involved in mitochondria, inflammation, and transcription regulation were found to be altered in MetS (17).

Identifying miRNA and mRNA cargo of circulating EVs, and elucidating alternations imposed by disease status, could illuminate their potential as biomarkers of tissue and organ injury. However, how the presence of MetS affects the overall transcriptome of circulating EVs in human subjects and which pathways are primarily altered remains unknown. Therefore, we hypothesized that MetS is associated with altered mRNA and miRNA content in EVs isolated from systemic human plasma. To test this, we took advantage of high-throughput mRNA and miRNA sequencing to compare the gene and miRNAs profile between Lean- and MetS-human subjects.

## Materials and Methods

### Patient Population

MetS patients and healthy subjects (n=4 each group) were recruited in the First Hospital Affiliated to Jinan University (Guangdong, China). Our study followed the Declaration of Helsinki, was approved by the Institutional Research Ethics Committee, and written informed consent obtained from all subjects. All study participants were reviewed for medical history.

Inclusion criteria for MetS patients included older than 18 years and diagnosis of MetS, based on criteria presented by the International Diabetes Federation (18). The diagnosis of the MetS centered on obesity (body mass index[BMI] >30 kg/m2) and two or more of the following: abnormal lipids metabolism (HDL-cholesterol <50 mg/dl in females and <40 mg/dl in males, triglycerides ≥150 mg/dl), systolic blood pressure ≥130 mm Hg or diastolic blood pressure ≥85 mm Hg, previously diagnosed hypertension or type 2 diabetes or fasting glucose concentration ≥100 mg/dl. Exclusion criteria included cancer, heavy smoking, drug abuse, severe cardiac valvular diseases, any severe systemic diseases, or alcohol consumption in the past 3 months.

Inclusion criteria for healthy controls included overall health status, older than 18 years. Exclusion criteria for healthy controls included any significant disease, drug abuse, heavy smoking, or alcohol consumption in the past 3 months. None of these Lean or MetS subjects have been taking non-steroidal anti-inflammatory drugs (NSAIDs) or changed their exercise regimen within 3 months of enrolment.

Blood samples were collected under fasting condition for assessment of metabolic, renal, and liver functions following routine procedures in the clinical laboratories of the First Hospital Affiliated to Jinan University. Estimated glomerular filtration rate (eGFR) was calculated by the Modification of Diet in Renal Disease eGFR Equation (19).

### Blood EV Harvesting

EVs were isolated using the exoRNeasy Serum/Plasma (Qiagen cat# 77044) assay, followed by RNA isolation using the miRNeasy serum/plasma advanced kit (Qiagen cat# 217204), as per manufacturer’s directions. Briefly, thrombin was added to the plasma, incubated for 5 minutes at room temperature, and centrifuged at 2,500*g* for 15 min. The supernatant was then mixed with precipitation buffer and incubated for 60 min at 4°C. Following centrifugation at 13,000*g* for 5 min, the pellet was resuspended and served for RNA isolation. The resuspended pellet was lysed, protein was precipitated and removed, isopropanol was added to the supernatant, and the sample loaded onto the column. Following three washes, RNA was eluted and stored at −80°C.

EVs were characterized post-isolation based on transmission electron microscopy and expression of EV markers (CD9, CD63, CD81) by Western blot, as previously published (10), and their concentrations determined using nanoparticle tracking analysis. In addition, their cargo was compared to typical EV markers listed in ExoCarta (http://www.exocarta.org), a web-based resource of exosomal cargo.

### mRNA Sequencing Analysis

RNA sequencing was performed and analyzed as described previously (10). RNA libraries were prepared according to the manufacturer’s protocol (TruSeq RNA Sample Prep Kit v2, Illumina, San Diego, USA) and loaded onto flow cells (8–10 pM) to generate cluster densities of 700,000/mm^2^ following the standard protocol. Then cells were sequenced on an Illumina HiSeq 2000 using TruSeq SBS kit version-3 and HCS v2.0.12 data collection software, with data analyzed using the MAPRSeq v.1.2.1 system and the Bioinformatics Core standard tool. The mRNA-Seq data were analyzed using CAP-miRSeq v1.1, normalized, and differential expression analyzed using edgeR 2.6.2. Gene expression was normalized to 1 million reads and corrected for gene length (reads per kilobasepair per million mapped reads). DEseq analysis was performed and mRNAs showing fold-change >1.4 between the groups, and p<0.05 were considered upregulated, whereas those with fold-change <0.7 and p<0.05 were considered downregulated. Functional annotation clustering analysis was performed using DAVID 6.7 database (http://david.abcc.ncifcrf.gov/) to obtain a ranking of primary gene ontology categories for upregulated and downregulated mRNAs.

### miRNA Sequencing and Data Analysis

EVs total RNA libraries were prepared using QIAseq Stranded Total RNA Kit. MiRNA sequencing libraries prepared with QIAseq miRNA Library Kit were sequenced with an Illumina NGS system (MiSeq Personal Sequencer, NextSequence500, HiSeq-1000, HiSeq-1500, HiSeq- 2000, HiSeq-2500, and GaIIx). The data were analyzed with CLC (Biomedical) Genomics Workbench. Starting with unaligned FASTQs, the workflow generates aligned BAMs and then both raw and normalized known mature miRNA expression counts. The R-based tool from Bioconductor, edgeR2.6.2 was used to perform DEseq analysis to identify miRNAs enriched in MetS-EVs compared to Lean-EVs (fold-change >2.0 or fold-change <2 and p<0.05). TargetScan 7.1 and miRWalk 2.0 were used to predict target genes of significantly upregulated and downregulated miRNAs. Subsequent functional annotation-clustering analysis utilized the PANTHER (http://www.pantherdb.org/) and DAVID 6.7 database. Gene targets of miRNAs enriched in Lean- and MetS-EVs were compared on different categories, including cellular component, molecular function, biological process, and biological pathway.

### Validation of miRNA and mRNA Expressions

To validate expression of representative mRNAs and miRNAs in circulating human EVs, the expression of several candidate RNAs was confirmed by quantitative polymerase chain reaction (qPCR). Total RNA was isolated from plasma-derived EVs, and probed with primers (Ribobio, Guangzhou, Guangdong Province, China; GZP2020081200495, GZP2020081200501, GZP2020081200497, GZP2020081200484, GZP2020081200483, and GZP2020081200482). All results were adjusted by GAPDH.

### Integrated mRNA/miRNA Analysis

Target prediction analysis of dysregulated miRNAs was performed using miRWalk 3.0 (http://mirwalk.umm.uni-heidelberg.de/) and target genes that overlapped with those mRNAs dysregulated in MetS-EVs analyzed using Venny 2.1 (https://bioinfogp.cnb.csic.es/tools/venny/).

### Statistical Analysis

Statistical analysis was performed using JMP 14.0 (SAS Institute, Cary, NC). Data are expressed as mean ± standard deviation. The Shapiro–Wilk test was used to test for deviation from normality. Normally distributed data were compared using unpaired Student’s t-test and ANOVA. Nonparametric tests (Wilcoxon and Kruskal Wallis) were used when data did not follow a Gaussian distribution. Statistical significance was accepted if p ≤ 0.05.

## Results

### Systemic Characteristics of Lean and MetS Patients

**Table 1** shows the demographic, clinical, and laboratory characteristics of the study patients. BMI, systolic, and diastolic blood pressures were all markedly higher in the MetS compared to Lean subjects, whereas age and sex were similar between the groups. Low-density lipoprotein, fasting blood sugar, C-peptide, insulin, and hemoglobin A1C levels were also higher in MetS compared to Lean, underscoring development of MetS. Elevated blood urea nitrogen (BUN) and eGFR were consistent with development of hyperfiltration that characterizes obese individuals, and plasma renin activity (PRA) was increased. Elevated alanine aminotransferase (ALT), aspartate aminotransferase (AST), and alkaline phosphatase (ALP) indicated early liver injury in the MetS group, whereas a higher white blood cell count was consistent with systemic inflammation.

**Table 1:** Clinical, laboratory, and demographic data of Lean and Mets patients (n = 4 each).

Parameters	Lean	Mets
Age (years)	24.5 (21–29)	29.3 (24–32)
Sex (female/male)	2/2	2/2
Duration of MetS (years)	–	5.25 ± 1.71
Body mass index (Kg/mm2)	19.1 ± 0.9	62.2 ± 14.2*
Systolic blood pressure (mmHg)	113 ± 11.9	150.3 ± 9.7*
Diastolic blood pressure (mmHg)	63.8 ± 5.9	95 ± 4.5*
Hemoglobin A1C (%)	5.4 ± 0.2	7.2 ± 0.8*
Total cholesterol (mmol/l)	4.5 ± 0.4	5.1 ± 0.5
Triglycerides (mmol/l)	1.75 ± 0.2	2.42 ± 0.8
High-density lipoprotein (mmol/l)	0.96 ± 0.1	0.97 ± 0.2
Low-density lipoprotein (mmol/l)	1.6 ± 0.3	3.1 ± 0.6*
Blood urea nitrogen (mmol/l)	3.7 ± 0.5	5.4 ± 1.6*
eGFR (ml/min/1.73m2)	129.3 ± 39.1	193.4 ± 7.4*
Fasting blood sugar (mmol/l)	4.9 ± 0.4	8.2 ± 1.6*
Insulin (mIU/L)	14.0 ± 4.4	42.4 ± 16.1*
C-peptide (ng/ml)	2.5 ± 0.6	6.0 ± 0.8*
Alanine aminotransferase (U/L)	29 ± 7.0	100 ± 23.5*
Aspartate aminotransferase (U/L)	25.5 ± 7.4	86.5 ± 29.4*
Alkaline phosphatase (U/L)	63.5 ± 5.2	85.3 ± 15.4*
White blood cells *10^9/L	4.15 ± 1.1	8.28 ± 2.3*
Plasma renin activity (ng/ml/h)	0.6 ± 0.1	3.5 ± 0.5*

*P < 0.05 vs Lean.

eGFR, estimated glomerular filtration rate.

### Characterization of Lean- and MetS Circulating EVs

**Figure 1:**
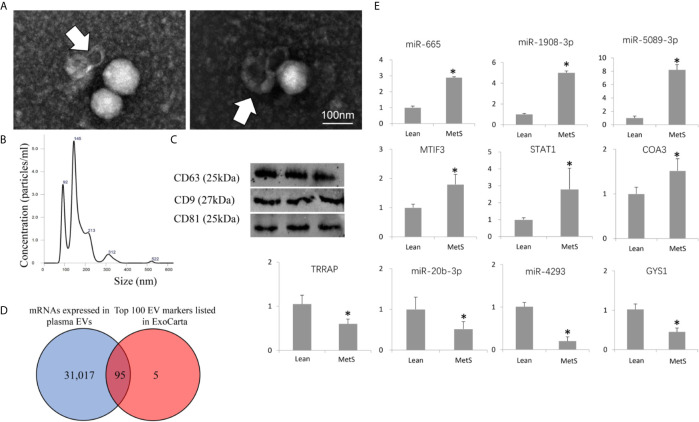
Characterization of circulating extracellular vesicles (EVs) and validation of dysregulated miRNAs. **(A)** Transmission electron microscopy (negative staining) showing EV clusters (arrows) with the classic “cup-like” morphology. **(B)** Size distribution of isolated EVs revealed a composition of about 2/3 small microvesicles (~145 nm in size) and 1/3 exosomes (~92 nm). **(C)** Western blotting analysis showing that isolated EVs expressed common EV markers (CD9, CD63, and CD81). **(D)** Venn diagram showing that 95 mRNAs of the top 100 EV markers listed in ExoCarta were found in the isolated EVs. **(E)** Expression of candidate mRNAs and miRNAs (qPCR) was concordant with miRNA-seq and mRNA-seq results. *p ≤ 0.05 vs. Lean.

Transmission and scanning electron microscopy demonstrated that the plasma contained EVs with the traditional “cup-like” morphology (**Figure 1A**). Circulating EVs expressed typical EV (CD9, CD81, and CD63) markers, and NTA demonstrated a typical size distribution of microparticles and exosomes (**Figures 1B, C**). Furthermore, of the top 100 conventional EV markers listed in ExoCarta, 95 mRNAs were identified in isolated EVs (**Figure 1D**), confirming their nature.

### EV mRNAs Cargo

**Figure 2:**
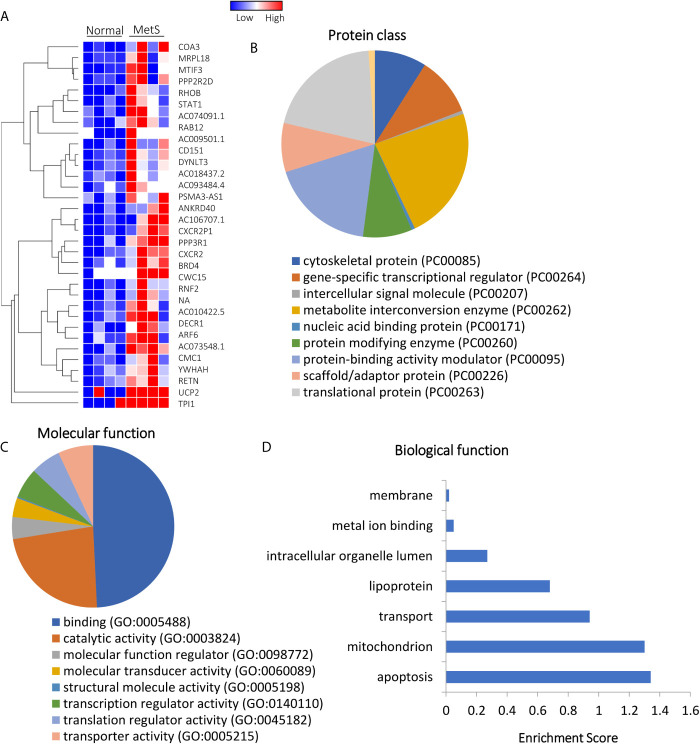
Upregulated mRNAs in Lean and MetS plasma-EVs. **(A)** Heat-map showing 32 upregulated mRNAs in MetS compared with Lean plasma-EVs. Panther analysis showed protein class **(B)** and molecular function **(C)**. **(D)** Enrichment of functional pathways of the 32 upregulated genes using DAVID 6.7.

**Figure 3:**
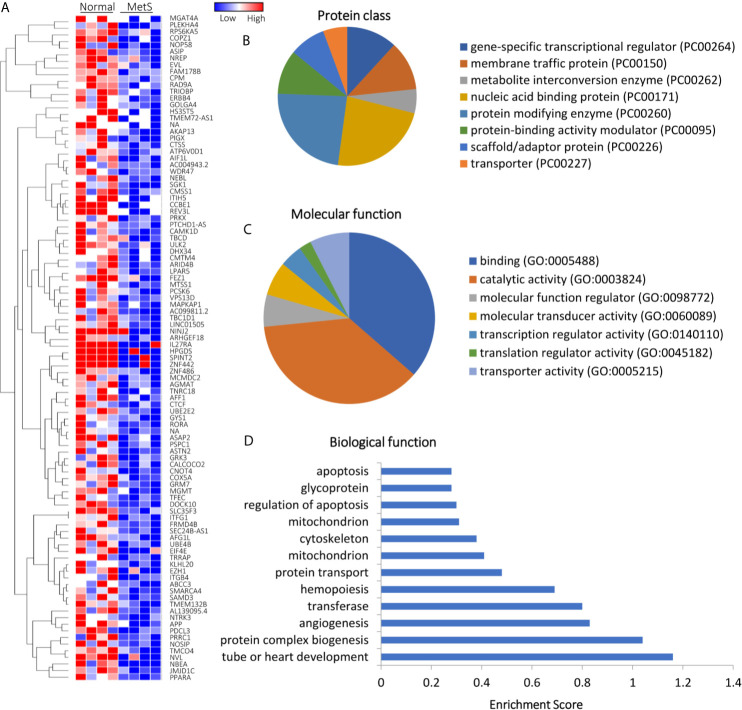
Downregulated mRNAs in Lean and MetS plasma-EVs. **(A)** Heat-map showing top 100 downregulated mRNAs in MetS compared with Lean plasma-EVs. Panther analysis showed protein class **(B)** and molecular function **(C)**. **(D)** Enrichment of functional pathways of the top 100 downregulated genes using DAVID 6.7.

Of all annotated genes (n=32,392), mapping of RNA reads revealed 32 genes (0.1%) upregulated in circulating MetS- compared with Lean-EVs (**Figures 2A** and **S1**). The genes encode for metabolite interconversion enzymes with protein binding and catalytic activity (**Figures 2B, C**). Functional analysis revealed that these proteins are primarily implicated in regulation of apoptosis (RHOB, PPP2R2D, DYNLT3), and mitochondrial function (MRPL18, MTIF3, UCP2), followed by transport and lipoproteins (**Figure 2D**). Contrarily, 1,446 (4.5%) mRNAs were downregulated in MetS compared to Lean-EVs. Functional annotation clustering analysis showed that those top 100 genes (**Figure 3A**) encode for nucleic acid binding and scaffold-adaptor proteins with binding and catalytic activity (**Figures 3B, C**), primarily involved in tube, vascular or heart development (NEBL, AKAP13, ERBB4), protein complex biogenesis (TRIOBP, TBCD, SMARCA4), and angiogenesis (APP, PRKX), followed by hemopoiesis, mitochondria, cytoskeleton, and regulation of apoptosis (**Figure 3D**).

### EV MicroRNAs Cargo

**Figure 4:**
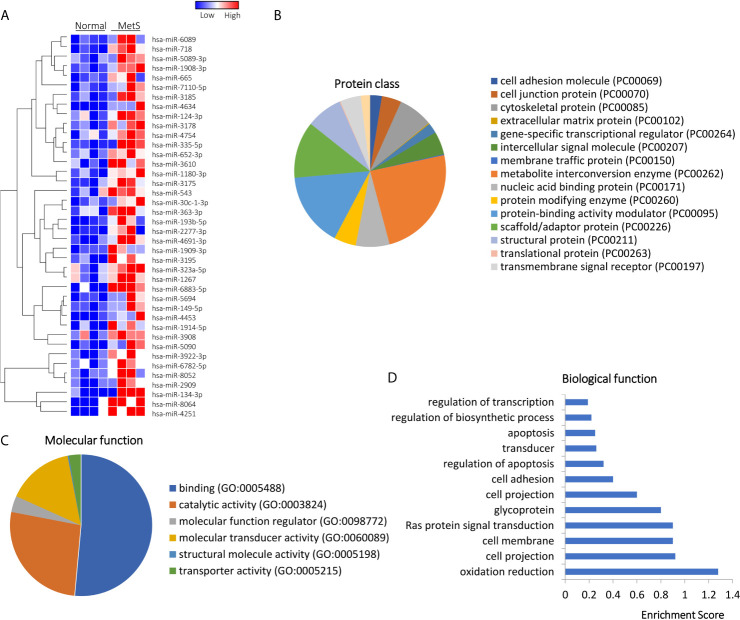
Upregulated miRNAs in Lean and MetS plasma-EVs. **(A)** Heat-map showed 40 upregulated miRNAs in MetS compared with Lean plasma-EVs. Panther analysis illustrated protein class **(B)** and molecular function **(C)**. **(D)** Enrichment of functional pathway of the 40 upregulated miRNAs target genes using DAVID 6.7.

**Figure 5:**
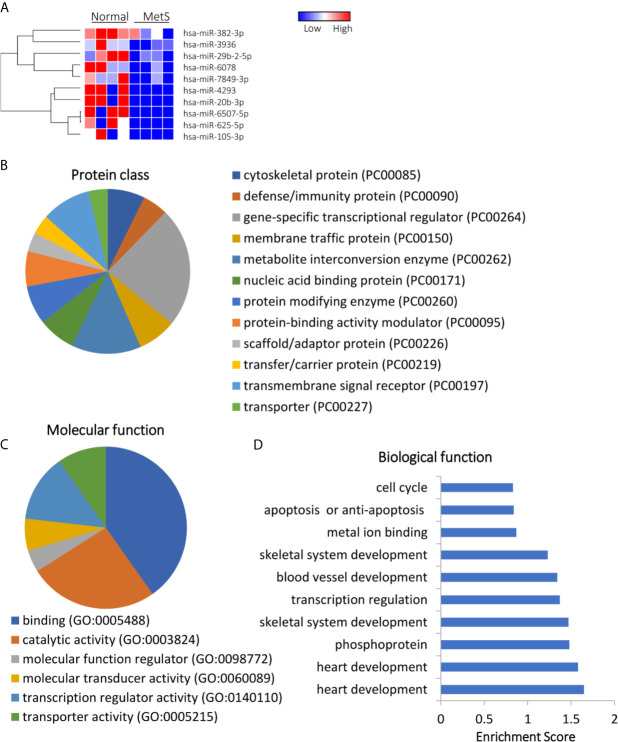
Downregulated miRNA profile in Lean and MetS plasma-EVs. **(A)** Heat-map showed 10 downregulated miRNAs in MetS compared with Lean plasma-EVs. Panther analysis depicted protein class **(B)** and molecular function **(C)**. **(D)** Enrichment of functional pathway of the 10 downregulated miRNAs target genes using DAVID 6.7.

Of 1,515 annotated miRNAs, 40 (2.6%) distinct miRNAs were selectively enriched in MetS EVs (**Figures 4A** and **S2**). Functional annotation clustering analysis showed that those upregulated miRNAs targeted genes encoding for cell junction proteins with binding activity (**Figures 4B, C**), primarily involved in redox regulation and oxidation-reduction (OXA1L, PNPT1, SDHC), cell structure (DCTN1, EFHC2, RHOA), and Ras protein signal transduction (BAD, RAF1, RELA), followed by regulation of apoptosis and transcription (**Figure 4D**). Contrarily, 10 miRNAs (0.7%) were downregulated in MetS-EVs compared with Lean-EVs (**Figure 5A**). The downregulated miRNAs targeted genes encode for gene-specific transcriptional regulators with binding and catalytic activity (**Figures 5B, C**), mostly implicated in heart and skeletal development (HEY2, SRF, NOTCH1), phosphoprotein (PPM1D, RPL26), and transcription regulation (RLF, TRIP4, ZNF45), followed by blood vessel development, apoptosis, and cell cycle (**Figure 5D**). qPCR confirmed that the expression pattern of several candidate mRNAs and miRNAs was similar to RNAseq results (**Figure 1E**).

### Integrated mRNA/miRNA Analysis

**Figure 6:**
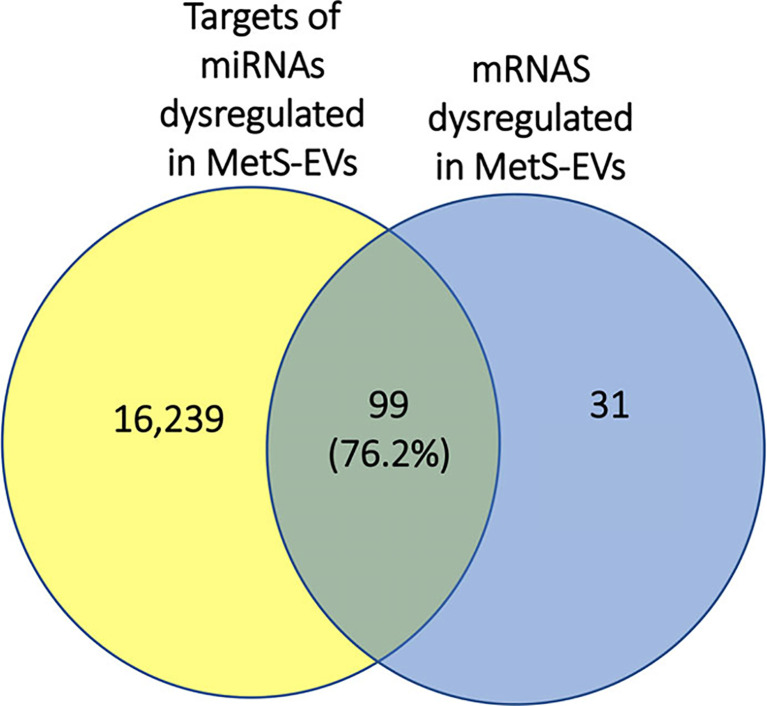
Representative Venn diagram showing that 76.2% of mRNAs dysregulated in MetS-EVs could be targeted by miRNAs dysregulated in MetS-EVs.

Integrated miRNA/mRNA analysis evaluated the interactions among dysregulated mRNAs and miRNAs. It revealed that 76.2% of mRNAs dysregulated in MetS-EVs were targets of miRNAs dysregulated in the same MetS-EVs (**Figure 6**).

## Discussion

Our study used high-throughput RNA-sequencing to interrogate mRNA and miRNA content of circulating EVs and explore the putative function of enriched or excluded genes and miRNAs within those EVs obtained from patients with MetS compared to lean controls. We demonstrated that MetS is associated with altered content of genes and miRNAs in human circulating EVs. Specifically, we found in MetS circulating EV upregulated genes involved in regulation of apoptosis, mitochondria, transport, and lipoproteins, but downregulated genes responsible for cardiovascular development and angiogenesis. MetS-EVs also showed upregulated miRNAs that target genes and proteins implicated in multiple cellular processes, including redox regulation and cell structure. Contrarily, miRNAs downregulated in MetS-EVs are capable of modulating proteins implicated in several cellular processes including heart, blood vessel, and skeletal development, transcription regulation, metal ion binding, apoptosis, and cell cycle. These observations suggest impaired cellular structure, function, and fate in MetS, which may blunt development or repair of blood vessels.

About one third of US adults and over a billion people globally have MetS (20). MetS fosters development of type 2 diabetes, lipid disorders, cardiovascular disease, hepatic steatosis, and other circulatory disorders (1, 2). Fundamental manifestations of MetS include insulin resistance and adipocyte dysfunction, which promote oxidative stress and chronic inflammation, and in turn damage in target tissues such as the kidney, liver, brain, and vasculature. In the kidney, MetS induces hyperfiltration and contributes to microvascular remodeling, podocyte injury, and mitochondrial dysfunction (21), whereas in the liver, non-alcoholic fatty liver disease is a manifestation of MetS (22). Congruently, we found that our patients with MetS showed elevated BUN, eGFR, ALT, AST, and ALP, indicating early renal and liver injury.

Circulating EVs are released from various tissues and organs, reflect the status of their parental cells, and may also mediate important processes in target cells (5). We found that several biological protein functions, including protein complex biogenesis and transport are targeted by dysregulated miRNAs within those EVs. In addition, MetS is a proinflammatory state associated with oxidative stress and apoptosis (19, 23), as also reflected in circulating EVs in our study. Biological functions targeted by upregulated miRNAs included redox regulation, which might be altered in parent cells that released those EVs to the systemic circulation. Interestingly, apoptosis and cell cycle are targeted by dysregulated miRNAs, consistent with our recent findings showing that MetS dysregulates in MSC-derived EVs miRNAs that regulate cellular senescence (24). In addition, MetS may alter angiogenesis and blood vessel development (25), which we found to be targeted by dysregulated miRNAs, suggesting that MetS might impair vascular reparative processes in response to ischemia or wound healing.

Mitochondrial dysfunction plays a role in MetS and advances as the disease progresses from insulin resistance to type 2 diabetes (26). Our previous studies have shown that obesity, as observed in our patients, impairs MSC mitochondrial structure and function, possibly mediated partly through miRNA-induced mitochondrial gene regulation, leading to increased oxidative stress (15, 27, 28). The current study extends our previous observations, demonstrating that MetS-EVs contain mitochondria-related mRNAs, which may reflect mitochondrial damage in the parent cells that released those EVs to the systemic circulation. Several mitochondrial genes upregulated in MetS-EVs included cytochrome oxidase assembly factor-3, a novel regulator of mitochondrial COX1 translation and cytochrome oxidase assembly (29), mitochondrial translational initiation factor-3, and signal transducer and activator of transcription. The product of this gene is required for recognition and regulation of translation initiation of mitochondrial mRNAs and for coordinated assembly of oxidative phosphorylation complexes *in vivo* (30). Likewise, the transcription factor signal transducer and activator of transcription regulates mitochondria-mediated oxidative stress response, PKCδ activation, and autophagy (31). Hence, our observations suggest mitochondrial dysfunction in MetS detectable in circulating EVs.

We found upregulation of genes linked to lipoprotein metabolism, consistent with the marked dyslipidemia in our MetS patients. Similarly, genes upregulated in MetS-EVs are implicated in apoptosis, including RHOB, PPP2R2D, dynein light chain Tctex-type 3, and C-X-C motif chemokine receptor-2 genes, which modulate specific apoptosis pathways (32). Apoptosis is a vital component of fundamental processes including normal cell turnover, proper development and functioning of the immune system, hormone-dependent atrophy, and others (33). RhoB, a Rho family GTPase, regulates cell cycle progression and is required for the apoptotic response of transformed fibroblasts to DNA damage (34). RhoB also contributes to cancer progression by regulating cell cycle progression, apoptosis, DNA damage responses, invasion, and migration. Recently, dynein light chain Tctex-type 3 has been reported to foster ovarian cancer through promoting cell proliferation, migration, and invasion (35). C-X-C motif chemokine receptor-2 has been found to promote anti-apoptosis, anti-senescence, and epithelial-to-mesenchymal transition of breast cancer cells, leading to enhanced metastasis and chemoresistance (36). Therefore, our findings suggest that MetS might impact cellular fate.

miRNAs play an essential role in regulating gene expression in several different physiological and pathophysiological conditions (15, 37), including metabolic diseases. In this study, many miRNAs were dysregulated in circulating EVs from MetS patients compared with healthy individuals. miRNAs upregulated in MetS plasma could be delivered to recipient cells and modulate cellular pathways. For example, miR-124, which participates in inflammation, autophagy, mitochondrial function, and neurotransmission (38), was selectively enriched in MetS EVs. Similarly, mir-149 that mediates inhibition of cell proliferation, migration, and invasion, and induces apoptosis, was also enriched in MetS-EVs (39). Taken together, these observations suggest that dysregulated miRNAs in circulating MetS-EVs might reflect imbalanced homeostasis and modulate cellular pathways.

Our study combined and comprehensively analyzed the mRNAs and miRNAs content in human circulating-EVs, and has a number of strengths. Using next-generation sequencing analysis, we identified differential mRNAs and miRNAs expression signatures in circulating EVs in MetS compared with Lean human subjects. These observations suggest that the mRNA and miRNA cargo of circulating EVs might represent novel biomarkers in MetS. However, further studies are needed to analyze different body fluids in chronic metabolic diseases to identify additional EV biomarkers (mRNAs and miRNAs) that can be used for diagnosis, prognosis, and therapeutics. In addition, further studies are needed to determine the relative contributions of the individual components of MetS (e.g., obesity, hypertension, hyperlipidemia, etc.), as well as the causal relationship between EV content, MetS, and the cellular damage that it is known to induce. Our study is limited by the small sample size, as is often used in mRNA- and miRNA-seq studies that produce large datasets (15, 24). Our patients were relatively young, arguing against a major role of aging in our changes in the cargo of circulating EVs. Lastly, MetS patients were extremely obese, so whether milder forms of obesity and MetS would lead to comparable alterations remains to be defined. Further studies are also needed to explore in detail genes and molecules that regulate pathogenic pathways in MetS, as well as techniques to blunt them.

## Conclusion

In summary, we found in human MetS modifies cargo of circulating EVs, which may in turn modulate several important cellular functions and fate, and potentially serve as key regulators, biomarkers, and targets in the progression and treatment of MetS. Genetic message related to mitochondrial function, apoptosis, angiogenesis, oxidative stress, and inflammatory pathways were dysregulated. Further studies are needed to determine whether these changes in circulating EVs could be delivered to recipient cells and modulate cellular pathways.

## Data Availability Statement

The datasets presented in this study can be found in online repositories. The names of the repository/repositories and accession number(s) can be found below: www.ncbi.nlm.nih.gov/bioproject/672664, RPJNA672664.

## Ethics Statement

The studies involving human participants were reviewed and approved by the First Hospital Affiliated to Jinan University Institutional Research Ethics Committee (Guangdong, China). The patients/participants provided their written informed consent to participate in this study.

## Author Contributions

YL participated in the experimental design, data collection and assembly, data analysis, and interpretation, manuscript writing. YM participated in the collection and assembly of data, financial support, data analysis, and interpretation. AW participated in the data analysis and interpretation, manuscript editing. AE participated in the conception and design, data collection and analysis, manuscript editing. LL participated in the conception and design, financial support, manuscript editing. All authors contributed to the article and approved the submitted version.

## Funding

This study was partly supported by NIH grant numbers DK120292, DK122734, AG062104, DK106427, and DK122137, Science and Technology Program of Guangzhou 202102010133 and the research Foundation of Shenzhen JCYJ20190808095615389.

## Conflict of Interest

LL is an advisor to AstraZeneca, Butterfly Biosciences, and Janssen Pharmaceuticals.

The remaining authors declare that the research was conducted in the absence of any commercial or financial relationships that could be construed as a potential conflict of interest.

## Publisher’s Note

All claims expressed in this article are solely those of the authors and do not necessarily represent those of their affiliated organizations, or those of the publisher, the editors and the reviewers. Any product that may be evaluated in this article, or claim that may be made by its manufacturer, is not guaranteed or endorsed by the publisher.

## Abbreviations

MetS, metabolic syndrome; EVs, extracellular vesicles; miRNA, micro-RNA; eGFR, estimated glomerular filtration rate; ALT, alanine aminotransferase; AST, aspartate aminotransferase; ALP, alkaline phosphatase; PRA, plasma renin activity. MSCs: mesenchymal stem cells.
