# Supplementary material for: Metabolic Syndrome Is Associated With Altered mRNA and miRNA Content in Human Circulating Extracellular Vesicles
Source: Front Endocrinol (Lausanne). 2021 Aug 12;12:687586. doi: 10.3389/fendo.2021.687586 (PMC8387871; doi:10.3389/fendo.2021.687586)
Supplement: Supplementary file 1 [file DataSheet_1.pdf]

# Metabolic Syndrome is associated with altered mRNA and miRNA Content in Human Circulating Extracellular Vesicles

Running Title: modified mRNAs and miRNAs in MetS Plasma

Authors: Yongxin Li[1,2], Yu Meng[3,4 *], Xiangyang Zhu[2], Andre Van Wijnen[5], Alfonso Eirin[2], Lilach O. Lerman[2 *]

[1]Department of Vascular Surgery, the Affiliated Hospital of Qingdao University, Qingdao, 266000, PR China

[2]Division of Nephrology and Hypertension, Mayo Clinic, Rochester, MN 55905, USA

[3]Central laboratory, the Fifth Affiliated Hospital of Jinan University, Heyuan 517000, PR China

[4]Department of Nephrology, the First Affiliated Hospital of Jinan University, Guangzhou, 510630, PR China

[5]Departments of Orthopedic Surgery, Biochemistry and Molecular Biology, Mayo Clinic, Rochester, MN 55905, USA

**Fig S1.** Heat Map and volcano plot showing 32 (0.1%) mRNAs upregulated and 1,446 (4.5%) mRNAs downregulated in MetS compared to Lean-EVs.

**Fig S2.** Heat Map and volcano plot showing 40 (2.6%) miRNAs upregulated and 10 (0.7%) mRNAs downregulated in MetS compared to Lean-EVs.

Fig. S1

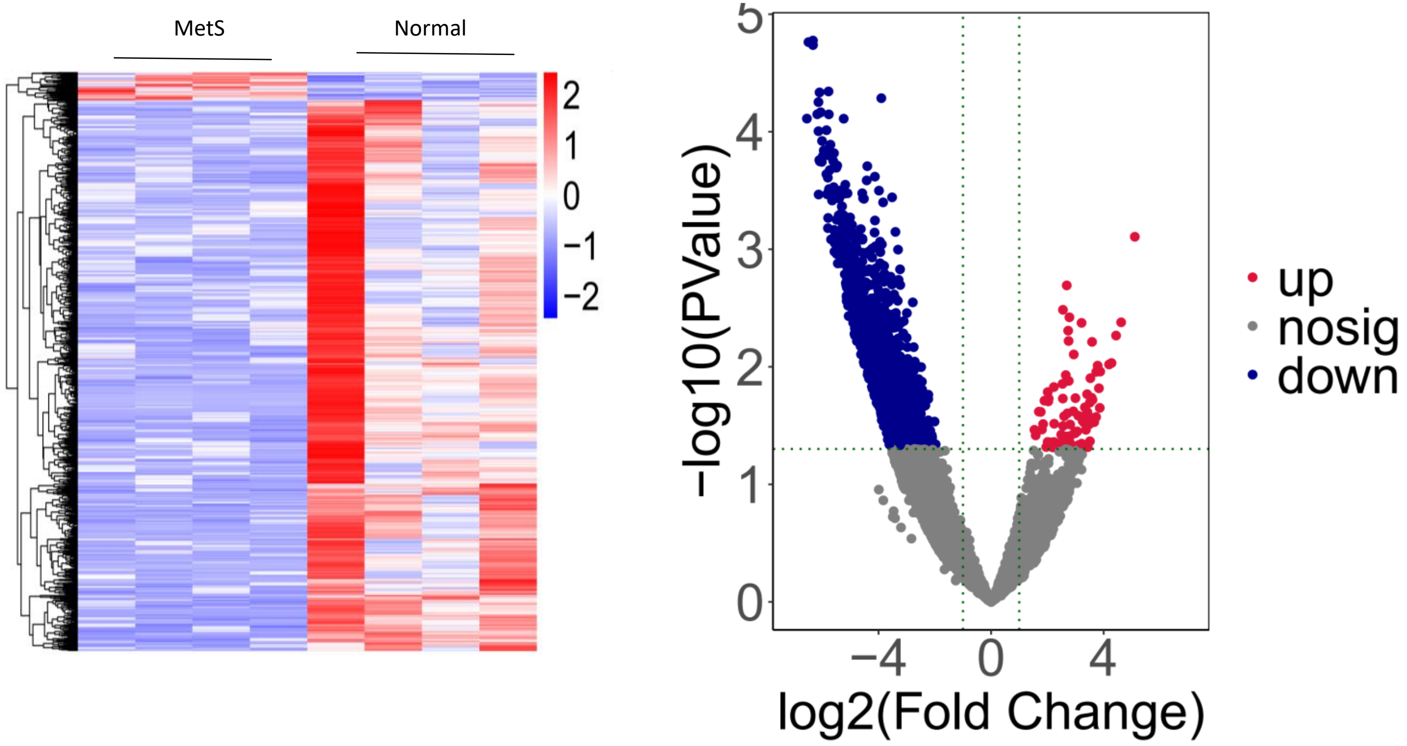

MetS
Normal
2
1
0
−1
−2
5
4
3
2
1
0
−log10(PValue)
−4
0
4
log2(Fold Change)
up
nosig
down

Fig. S2

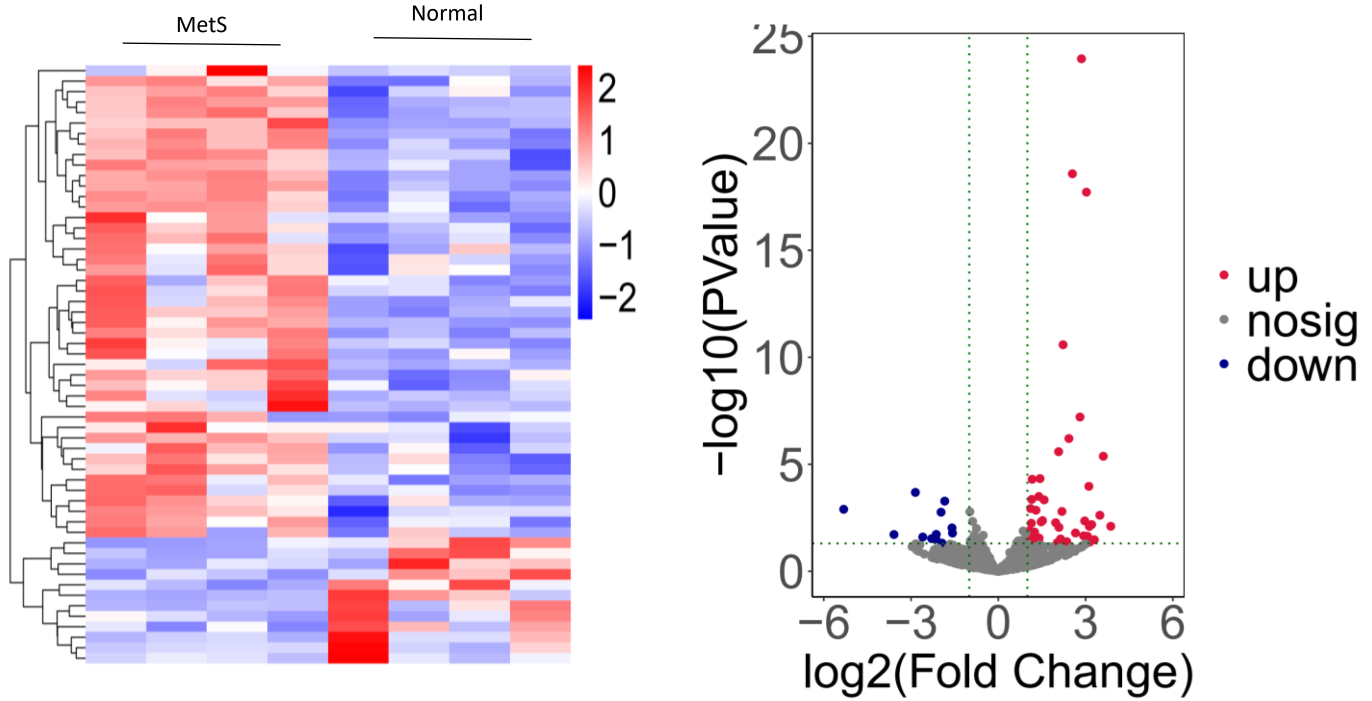

MetS
Normal
2
1
0
−1
−2
25
20
15
10
5
0
−log10(PValue)
−6
−3
0
3
6
log2(Fold Change)
up
nosig
down
